# The Evolution of Prognostic Factors in Multiple Myeloma

**DOI:** 10.1155/2017/4812637

**Published:** 2017-02-21

**Authors:** Amr Hanbali, Mona Hassanein, Walid Rasheed, Mahmoud Aljurf, Fahad Alsharif

**Affiliations:** King Faisal Specialist Hospital and Research Center, Riyadh, Saudi Arabia

## Abstract

Multiple myeloma (MM) is a heterogeneous hematologic malignancy involving the proliferation of plasma cells derived by different genetic events contributing to the development, progression, and prognosis of this disease. Despite improvement in treatment strategies of MM over the last decade, the disease remains incurable. All efforts are currently focused on understanding the prognostic markers of the disease hoping to incorporate the new therapeutic modalities to convert the disease into curable one. We present this comprehensive review to summarize the current standard prognostic markers used in MM along with novel techniques that are still in development and highlight their implications in current clinical practice.

## 1. Introduction

Multiple myeloma (MM) is a heterogeneous hematologic malignancy involving the proliferation of plasma cells derived by different genetic events contributing to the development, progression, and prognosis of this disease. Despite improvement in treatment strategies of MM over the last decade, the disease remains incurable in most cases, although in recent years overall survival of patients has been significantly increased. All current efforts are focused on the development of novel diagnostic and therapeutic modalities hoping to convert the disease into a curable one. Over the last 15 years, new techniques in prognostic markers and novel imaging modalities became available.

Risk stratification of MM is essential for understanding the prognosis and modifications of therapeutic modalities. Patients with MM who are stratified as high risk, such as those with 17p13 deletion, generally have poor outcome with current treatment strategies and all efforts currently are focused on establishing alternative strategies for management of such patients. For the low-risk patients, they have at least 50% chance of surviving more than 10 years.

Our aim of this review is to summarize the current standard prognostic markers used in MM along with novel techniques that are still in development and highlight their implications in current clinical practice.

The prognostic factors of MM will be divided into 4 major sections:

1. Risk Stratification, which includes Staging of MM, Plasma Cell Labeling index (PCLI), Cytogenetics and Gene Expression Profiling (GEP)
2. Monitoring of Response Tools, which includes Serum-Free Light Chain Assay, serum Heavy/Light Chain (HLC) Assay (Hevylite™), and Advanced Imaging Modalities.
3. Minimal Residual Disease (MRD) Monitoring Methods, which includes Circulating Plasma Cells, MRD Monitoring in General, and the Value of Depth of Response
4. Novel Prognostic Markers

## 2. Risk Stratification

### 2.1. Staging of MM

Determining the prognosis in MM requires the knowledge of tumor and host factors. Work on stratifying MM into different stages started in the 1960s and early 1970s when a number of clinical and laboratory parameters were identified, including hemoglobin level, serum calcium, serum creatinine, and severity of bone lesions [1, 2]. In 1975, Durie and Salmon [3] developed a Durie-Salmon Staging (DS) system as a prognostic model using the following parameters that predicted myeloma cell tumor burden: hemoglobin level, serum calcium level, the number of bone lesions on bone X-ray, and the level and type of monoclonal protein.

Durie-Salmon staging system for multiple myeloma (see [3, 4]) is as follows.

*Stage I. *Low cell mass is <0.6 × 10^12^ cells/m^2^ plus all of the following:

- Hgb > 10 g/dL
- Serum IgG < 5 g/dL
- Serum IgA < 3 g/dL
- Normal serum calcium
- Urine monoclonal protein excretion < 4 g/day
- No generalized lytic bone lesions.

*Stage II.* Intermediate cell mass is neither stage I nor stage III.

*Stage III*. High cell mass is >1.2 × 10^12^ cells/m^2^ plus one or more of the following:

- Hgb < 8.5 g/dL
- Serum IgG > 7 g/dL
- Serum IgA > 5 g/dL
- Serum calcium > 12 mg/dL (3 *μ*mol/L)
- Urine monoclonal protein excretion > 12 g/day
- Advanced lytic bone lesions

Stage III is subclassified as IIIA or IIIB based on serum creatinine:

(A) Serum creatinine < 2 mg/dL (177 *μ*mol/L)
(B) Serum creatinine ≥ 2 mg/dL

DS system was adopted as a standard method for MM staging for many years and it became the most commonly used prognostic scheme in patients with newly diagnosed MM. The drawbacks of this system included the following: it focuses on variables correlate with myeloma mass and it does not take into account the biologic variability of the disease. Also, one of the important elements of DS system is the number of lytic lesions seen on skeletal survey, which is operator dependent. Since then, several other staging systems have been proposed using other known prognostic factors, including C-reactive protein albumin and plasma cell labeling index [5–8], but the one that gained wide acceptance was the international staging system (ISS) that was published in 2005 [4]. ISS is a simple staging system that is based on the serum beta-2 microglobulin (S*β*2M) and albumin.

International staging system for myeloma (see [4]) is as follows:

  Stage 1: *β*2M < 3.5 and ALB ≥ 3.5
  Stage 2: ALB < 3.5 and *β*2M < 3.5; ALB < 3.5; or *β*2M 3.5–<5.5
  Stage 3: *β*2M ≥ 5.5

where *β*2M is serum *β*2 microglobulin in mg/dL and ALB is serum albumin in g/dL.

The ISS evolved from a statistical model focusing on survival duration [4]. The ISS is a major improvement over the DSS in that it separates patients into cohorts using easily measurable, objective, and reproducible parameters [9]. The major criticism of ISS was the lack of the use of known other prognostic markers in MM including cytogenetics abnormalities (CA) and LDH. In 2015, Palumbo et al. [10] published revised international staging system (ISS-R) which combined ISS with CA and LDH as follows.

Revised international staging system (see [10]) is as follows:

  Stage I: ISS I, standard risk by FISH and normal LDH
  Stage II: not R-ISS I or III
  Stage III: ISS III, either high risk by FISH or high LDH

where (i) high risk by FISH is presence of del(17p) and/or translocation t(4;14) and/or t(14;16), (ii) standard risk by FISH is no high-risk chromosomal abnormalities, (iii) normal LDH is serum LDH < the upper limit of normal, and (iv) high LDH is serum LDH > the upper limit of normal.

ISS-R is proving to be a powerful prognostic staging system, but currently its use in practical practice is limited and it is used primarily for risk stratification of patients in clinical trials.

### 2.2. Plasma Cell Labeling Index

MM is characterized by proliferation of monoclonal plasma cells (PCs) in the bone marrow. There are certain characteristics of this proliferation that correlate with prognosis of MM, including plasma cell labeling index (PCLI), circulating plasma cells, and plasmablastic morphology.

PCLI is a measure of marrow plasma cells in S phase of the cell cycle, which provides a good estimate of the proliferative capacity of the malignant clonal plasma cells [11]. In 1993, Greipp et al. demonstrated that PCLI and B2M measured at diagnosis are independent prognostic factors in MM [12]. This was confirmed in other studies, including the study by Steensma et al., which demonstrated that high PCLI in patients with apparently stable, plateau phase MM is an adverse parameter that may predict a short time to disease progression and death [13]. Another study by Li et al. showed that PCLI was higher among patients with del (13q14), and patients with a high PCLI had a short time to disease progression [14]. Currently PCLI is rarely used because of the availability of more practical prognostic methods.

### 2.3. Cytogenetics

MM is a malignancy of plasma cells which develops through genetic aberrations, epigenetic changes, and the bone marrow microenvironment interaction. In the past decade, nonrandom chromosomal aberrations such as t(4;14), t(14;16), t(14;20), amp1q21, and del 17p have been shown to be associated with poor prognosis, and moreover, recent progress in genome-wide deep sequencing studies revealed mutations and intratumor subclonal heterogeneity which may explain the clinical phenotype and therapeutic resistance.

#### 2.3.1. t(4;14)

The prognostic significance of t(4;14) as detected by RT-PCR on BM and PB samples of 208 patients with MM and 52 patients with monoclonal gammopathy of undetermined significance (MGUS) was assessed. The results showed that the presence of this translocation is associated with poor survival (*P* = 0.006) and poor response to first-line chemotherapy (*P* = 0.05) [15]. At Mayo Clinic, in a series of 238 patients studied between 1990 and 2001, t(4;14) was determined in 153 patients, suggesting that high-dose therapy, as used to be in their practice, has minimal benefit for these patients with a median time to progression of only 8.2 months after stem cell therapy [16]. In another study, 19 patients with t(4;14) showed a good response to vincristine, doxorubicin, and dexamethasone (VAD) induction chemotherapy or pulsed dexamethasone alone, but early progression was common before HDT, with evident resistance to alkylating agents [17]. The results after a long term follow-up of 100 cases of MM with t(4;14), treated in IFM99 trials with tandem transplantation, revealed a heterogeneity in patients expressing t(4;14). They usually have similar overall response rates after both induction and HDT, to those achieved in patients without t(4;14). However, achievement of CR or VGPR after HDT in patients with t(4;14) was a powerful independent prognostic factor of outcome, with high risk of early relapse and dismal outcome in patients achieving only PR or less. In this study, the heterogeneity was not only related to response; the authors found that patients, who had b2-microglobulin of <4 mg/L and Hb level of ≥10 g/dL at diagnosis (45%), experienced improved survival after tandem transplant and benefited from HDT [18]. A clear separation of two groups of t(4;14) patients was reported by the Arkansas group using a 70-gene expression model [19]. The results of the 260 myeloma patients, enrolled in the GEM-2000 Spanish transplant protocol, reinforced the previous results from other series and confirmed that the presence of t(4;14) was sufficient for shortening MM patient survival [20]. The poor prognosis of patients with t(4; 14) may be in part due to its association with upregulation of the fibroblast growth factor receptor 3 (FGFR3). Data from the preclinical studies suggest that patients with increased expression of FGFR3 may benefit from the use of FGFR3 inhibitors [21]. Another interesting study showed that t(4;14) can be gained at time of relapse, which was observed in 14 out of 268 patients who did not express t(4;14) at diagnosis. Hypotheses that explain the acquisition of the t(4;14) at relapse include evolution of already present subclones or its acquisition during evolution [22].

#### 2.3.2. t(11;14)

A different translocation involving immunoglobulin heavy chain gene on chromosome 14, which is commonly associated with lymphomas, especially mantle cell lymphoma, was identified in 24 cases of multiple myeloma, by standard cytogenetic analysis; in most of these cases t(11;14)(q13;q32) was part of a complex karyotype and strong cyclin D1 overexpression by immunohistochemical stain [23]. In a large cohort including more than 350 myeloma patients, who participated in the Eastern Cooperative Oncology Group phase III clinical trial E9486, t(11;14)(q13;q32) was detected in approximately one-sixth of patients, and it was associated with a low serum monoclonal protein and plasma cell labeling index and is less likely to be hyperdiploid by DNA content analysis, which appeared to correlate with a better survival and prognosis in those patients [24]. The previous study, in addition to other studies, reported that the presence of t(11;14)(q13;q32) was always associated with small mature lymphoplasmacytoid morphology [24–26], and in more than 60% of the cases with CD20 expression [27]. Moreau et al. reported markedly improved long term survival in 26 patients with t(11;14)(q13;q32) after HDT [28], whereas patients with this translocation, who were treated within the Eastern Cooperative Oncology Group protocol with HDT, showed borderline improvement [24]. However, no effect on survival or time to progression was seen in patients with t(11;14)(q13;q32), treated with HDT at Mayo Clinic between 1990 and 2001 [16]. On the other hand, patients with t(11;14)(q13;q32) showed higher risk of extramedullary plasmacytoma- (EMP-) specific relapse compared to other cytogenetic abnormalities [29, 30] and a lower response rate, if they have EMP at presentation [31], which is supposed to be due to downregulation of CD56, which facilitate disease dissemination and malignant plasma cells extramedullary spread [32, 33]. In a further analysis of three hundred and four patients with newly diagnosed MM treated at Mayo Clinic between January 2004 and December 2012, who underwent serial cytogenetic evaluations, patients with t(11;14) showed an increased cytogenetic stability during the follow-up, with decreased odds of cytogenetic evolution (odds ratio (OR) = 0.22, 95% confidence interval (CI) = 0.09–0.56, *P* = 0.001) [34]. In contrast, Kaufman and colleagues reported inferior overall survival of patients with t(11;14) when compared with the classical standard risk patients in their cohort, which included 409 patients treated with HDT following doublet or triplet novel agent induction [35].

#### 2.3.3. t(14;16)

The data about t(14;16) are conflicting; on a retrospective analysis of over 1000 myeloma patients, the 32 patients with t(14;16) did not show any survival difference from patients lacking this translocation, and it was not proved to be an independent prognostic factor on multivariate analysis [50], while some studies reported that t(14;16) have a negative impact on prognosis [51, 52].

#### 2.3.4. Chromosome 13 Deletions

Chromosome 13 deletions either partial or complete detected by metaphase cytogenetics (CG) proved to have poor prognostic impact on patients with MM [53]. In subsequent studies, the rate of del(13q) detection was increased 2 to 3 times using interphase fluorescence in situ hybridization (FISH), but it remains an independent adverse prognostic [54–57]. In a further study that included 238 patients treated with HDT patients who expressed 13q del alone by FISH did not have a significantly shorter overall survival, but the presence of both 13q del and t(4;14) together had a significant adverse effect on outcome [58]. In addition, the presence or absence of del(13q14) did not seem to affect overall response to single agent bortezomib in 62 patients with relapsed/refractory MM [59].

#### 2.3.5. 17p13 Deletion

TP53 gene is located at 17p13; deletion of 17p13 is expressed in up to 11% of newly diagnosed myeloma patients. TP53 mutation, a well-known poor prognostic factor in many cancers, has also a strong correlation with poor outcome and resistance to therapy in patients with MM, less frequently expressed at diagnosis, but it becomes more detected at relapse or with advanced disease [60–62]. The work done by Lodè and colleagues showed that TP53 mutations are exclusively associated with del(17p); by sequencing for TP53 gene in 92 newly diagnosed myeloma patients, 37% of 54 patients with del(17p) have mutations of the TP53 gene (63% are homozygous), while none of the patients without del(17p) expressed TP53 mutation [60]. It is evident that the negative prognostic impact of del(17p) is demonstrated when at least 60% of plasma cells have it [63].

#### 2.3.6. Chromosome 1 Abnormalities

Chromosome 1 abnormalities are frequently detected in MM [31]; del 1p lead to loss of tumor suppressor genes and emerged as a poor prognostic factor in myeloma [64, 65]. The adverse prognostic role was confirmed in a study, which included 15 patients with del 1p; associated 13 q del was detected in 10 out of the 15 patients. Del 1p did not affect PFS in these patients after HDT and Autotransplant [66]. In addition, the role of chromosome 1 abnormalities was investigated in elderly patients (>65 years) enrolled in a phase III randomized clinical trial comparing VMP versus VMPT-VT; the abnormalities are when thalidomide appears to have a detrimental effect in elderly patients with newly diagnosed MM and abnormal chr1, while bortezomib can overcome its negative prognostic impact [67].

#### 2.3.7. Gene Expression Profile (GEP)

Several studies tried to identify molecular subgroups of multiple myeloma, using gene expression profiling on purified by CD138+ plasma cells. A study done in the University of Arkansas for Medical Science (UAMS), using plasma cells (PCs) from 74 newly diagnosed myeloma patients, 5 with monoclonal gammopathy of undetermined significance (MGUS), and 31 healthy volunteers (normal PCs), identified 4 distinct subgroups of MM (MM1, MM2, MM3, and MM4), ranging from MM1 that is more like normal PCs and MGUS, whereas MM4 showed more poor prognostic features as abnormal karyotype and high serum b2-microglobulin levels [68]. In a later study from (UAMS), they defined 7 subgroups rather than 4, using samples from over 400 newly diagnosed myeloma patients and more specific genes such as c-MAF and MAFB, CCND1, CCND3, ASS, IL6R, MMSET, FGFR3, CCNB2, FRZB, and DKK1. They described the UAMS classification 7 clusters, CD-1, CD-2, MS, MF, HY, PR, and LB [69]. Three novel subsets of multiple myeloma were identified, using data of the 320 newly diagnosed myeloma patients included in the Dutch-Belgian/German HOVON-65/GMMG-HD4 trial, in addition to 7 subgroups described in the 2006 UAMS classification, which were NF*κ*B, CTA, and PRL3 clusters [70].

Myeloma can be roughly divided into two equal disease entities: hyperdiploid multiple myeloma (H-MM) and Nonhyperdiploid multiple myeloma (NH-MM). A gene expression profiling study was conducted at Mayo Clinic trying to characterize the molecular profile of H-MM. Four nonoverlapping clusters were identified, each with distinct clinical and biological features, including a subgroup with a poor prognosis and a subgroup that responds fairly well to bortezomib [71].

The 15 most stable genes associated with survival from the 7,508-gene set used in the IFM 99 trials were used to stratify myeloma patients included in the trials into low-risk and high-risk groups; the authors concluded that high-risk patients have a 6.8-fold increased risk of death compared with low-risk patients (95% CI, 3.92 to 11.73; *P* < 0.001), with more than 90% survival rates for low-risk group and less than 50% for high-risk group at 3 years [72].

Another gene signature called EMC-92-gene signature was generated from gene expression profile used in the HOVON65/GMMG-HD4 trial. The performance of the EMC-92-gene signature was validated in newly diagnosed and relapsed myeloma patients, and it was proved to be independent of other prognostic factors on multivariate analysis. In addition, it was reported to be the best compared to other used signatures [73]. In another study done by Kuiper et al., they evaluated twenty risk markers, including t(4;14) and deletion of 17p (FISH), EMC92, and UAMS70 (GEP classifiers), and ISS. Their results showed that the EMC92-ISS combination is the strongest predictor for overall survival, resulting in a 4-group risk classification. The median survival was 24 months for the highest risk group and 47 and 61 months for the intermediate risk groups, and the median was not reached after 96 months for the lowest risk group [74].

### 2.4. Risk Stratification Models

Several risk stratification models have been developed for prognostication of MM patients. The most widely used are shown in Table 1.

## 3. Monitoring of Response Tools

### 3.1. Serum Light Chain Assay

Standard work-up of newly diagnosed MM includes assessment of both serum and urine for monoclonal protein. These biological markers have also proven to be essential in the disease progression detection and monitoring. A panel of members of the 2009 International Myeloma Workshop developed guidelines for standard investigative work-up of patients with suspected multiple myeloma. Both serum and urine should be assessed for monoclonal protein. Measurement of monoclonal protein both by the densitometer tracing and/by nephelometric quantitation is recommended, and immunofixation is required for confirmation. The serum-free light chain (sFLC) assay is recommended in all newly diagnosed patients with plasma cell dyscrasias [75]. Multiple studies have showed sFLCR to be a superior prognostic marker for plasma cell dyscrasias in contrast to M-spike. As an example, Dimopoulos et al. showed that, in patients with monoclonal gammopathy of undetermined significance (MGUS), the risk of progression in patients with an abnormal sFLC ratio (sFLCR) was significantly higher compared with patients with a normal ratio (hazard ratio, 3.5; 95% confidence interval [CI], 2.3–5.5; *P* < 0.001) and was independent of the size and type of the serum monoclonal (M) protein [75]. For patients with smoldering MM (SMM), Rajkumar et al. demonstrated that a high sFLCR > 100 is a predictor of imminent progression, and such patients may be considered candidates for early treatment intervention [76]. The prognostic value of sFLC was also seen in patients with solitary plasmacytoma of bone with significant higher progression to MM in patients with abnormal sFLCR [77]. In MM, abnormal sFLCR was shown to be an independent prognostic factor, with one study showing 5-year disease-specific survival of 82% in patients with sFLCR ≤ than the median compared to 30% in patients with sFLCR > the median (*P* = 0.0001) [78]. Because the half-life of FLC is <6 hours, FLC measurements at short sampling intervals allow real-time measurement of treatment-induced tumor kill and provide prompt indications of chemosensitivity [79].

### 3.2. Serum Heavy/Light Chain (HLC) Assay (Hevylite)

Immunofixation (IFE) is a standard method for detecting monoclonal immunoglobulins and characterizing its isotype. Recently clonality can also be determined by using immunoglobulin (Ig) heavy chain/light chain immunoassays (HLC), Hevylite. HLC separately measures in pairs light chain types of each intact Ig class generating ratio of monoclonal Ig/uninvolved polyclonal Ig concentrations [80]. Studies have shown that the HLC ratio (HLCR) is of prognostic significance in MM. According to results from a study by Koulieris et al. [81], high HLCR was associated with anemia, high serum FLCR, extensive bone marrow infiltration, and increased *β*2-microglobulin. In addition, increased HLCR and the presence of immunoparesis correlated with time to treatment initiation. Patients with high HLCR had a significantly shorter survival (*P* = 0.022). At the moment, HLC is considered novel immunoassays with multiple studies showing its utility in disease monitoring and outcome prediction in plasma cell dyscrasias. Its use is currently being cleared by the US Food and Drug Agency (FDA).

#### 3.2.1. Advanced Imaging Modalities

Imaging studies in MM include metastatic skeletal survey (MSS), computed tomography (CT), magnetic resonant imaging (MRI), and, more recently, positron emission tomography (PET) with fluorodeoxyglucose (FDG). MSS continues to be the standard diagnostic study in MM. Unfortunately, for MSS to detect bone destruction, the damage has to reach approximately 50% [82]. The national cancer center network (NCCN) MM panel recommends additional tests that may be useful under some circumstances. These include MRI and PET/CT [83]. Both MRI and PET scan are proven to give important information in patients with MM including detection of bone lesions, bone marrow infiltration, and disease monitoring posttherapy. A study by Baur-Melnyk et al. showed that patients without bone marrow infiltration have a significantly longer survival than patients with bone marrow infiltration in MRI at the time of diagnosis. However, even in stage I disease (Durie and Salmon) and negative X-ray films bone marrow infiltration in MRI may be detected in 29–50% of patients. Those patients typically show an earlier disease progression [84]. IMWG consensus considered MRI to be the gold-standard imaging technique for detection of bone morrow involvement [85]. The panel also discussed the prognostic value of MRI explaining that focal pattern on MRI gives prognostic information in symptomatic MM, and diffuse pattern also correlates with worse prognosis. Another study by Bredella et al. evaluated the value of FDG PET in the assessment of patient with MM and showed that FDG PET has sensitivity in detecting myelomatous involvement of 85% and specificity of 92%. FDG PET is able to detect bone marrow involvement in patients with MM and it is useful in assessing extent of disease at time of initial diagnosis, contributing to staging that is more accurate [86].

Despite numerous potential advantages of both MRI and PET-CT in MM, they are not yet the established gold standard for disease evaluation at diagnosis or at completion of therapy. Concerns with the serial use of these techniques exist due to the heterogeneity of visual criteria and the lack of consistency in the interpretation of results. Standardization of disease definitions for MRI and PET-CT imaging is needed to improve the specificity and positive predictive value of these tools [87].

Novel techniques can detect more lytic lesions compared to conventional radiography. Whole body, multidetector, low-dose computed tomography (WBLD-CT) is more sensitive for the detection of lytic lesions in myeloma compared to conventional radiography; it is very easy to perform (the examination is performed in 2 min or less), has a more accurate evaluation of areas with instability or at risk of fracture, and is superior regarding the planning for radiotherapy or surgical interventions [88].

For initial diagnosis of patients with multiple myeloma bone disease, use of an imaging test with a superior detection rate such as WBLDCT would find more lesions and presumably upstage patients, but definitive studies have yet to be completed defining the prognostic value of WBLDCT. WBLDCT can reliably exclude bone disease to confirm MGUS and complement laboratory monitoring. It remains unproven whether clinical benefit could be obtained by treating patients earlier or more aggressively based on WBLDCT findings. Nevertheless, recent data showing that early treatment of smoldering multiple myeloma leads to improved overall survival suggest that a more sensitive imaging method might help to detect lytic lesions and provide earlier treatment and thus improve survival [89]. Currently WBLDCT is considered a diagnostic tool, not a prognostic one.

## 4. Minimal Residual Disease (MRD) Monitoring Methods

### 4.1. Circulating Plasma Cells

Circulating PC detected by flow cytometry also is considered one of predictors of survival in patients with newly diagnosed MM. Nowakowski et al. studied the relationship between the number of circulating PCs in patients with newly diagnosed MM and survival and they concluded that it is an independent predictor of survival [90]. The increase in PC may be accompanied by morphological differences, like plasmablastic features, and can distinguish patients with a poor prognosis. Greipp et al. studied the prognostic significance of plasmablastic (PB) MM and the authors concluded that PB MM is a discrete entity associated with more aggressive disease and shortened survival [91]. However, since the prognostic values of these factors are not easily reproducible, they are not widely adopted [92].

### 4.2. Minimal Residual Disease (MRD)

It is currently well established that there is a direct relationship between depth of response and prolonged survival in MM [93, 94]. Still, the vast majority of patients who achieve complete response (CR) per the current definition criteria will eventually relapse. Because of that, the international Myeloma Working Group (IMWG), working on refining the criteria of CR in an effort to improve the outcome of the patients and in 2006, introduced normalization of sFLCs and absence of clonal PCs in BM biopsies by immunohistochemistry and/or immunofluorescence as additional requirements to define more stringent CR criteria [95]. Another CR definition that had emerged is molecular complete response (mCR), which is defined as absence of detectable disease by polymerase chain reaction (PCR) for Ig gene rearrangement [96]. Currently, the most sensitive approaches to detect MRD in MM include Multiparameter Flow Cytometry (MFC) and Ig allele-specific oligonucleotide-based quantitative PCR (ASO-PCR) [29]. The role of next generation sequencing (NGS) of Ig genes is emerging as a future sensitive tool to assess MRD. The sensitivity of these methods is comparable (MFC: 10^−5^ to 10^−6^, ASO-PCR: 10^−5^ to 10^−6^, NGS: 10^−6^) [97].

The prognostic value of MRD in MM has been explored in multiple studies. San Miguel et al. [98] studied the prognostic value of multiparametric immunophenotyping of PC compartment in patients with MM and found that ASCT provided a significantly greater reduction in the level of residual tumor PCs and with better recovery of normal PCs. The authors also found that patients in whom at least 30% of gated PCs had a normal phenotype after treatment had a significantly longer progression-free survival (60 months versus 34 months; *P* = 0.02). Paiva et al. on behalf of the GEM/PETHEMA cooperative study group [99] showed in MM patients who were treated with ASCT that median PFS (71 versus 37 months, *P* < 0.001) and median OS (not reached versus 89 months, *P* < .002) were longer in patients who were MRD negative versus MRD positive by multiparameter flow at day 100 after ASCT. Puig et al. compared ASO RQ-PCR with multiparameter FCM in patients with MM and found a significant correlation in MRD quantitation by both techniques (*r* = 0.881, *P* < 0.001), being reflective of treatment intensity. Patients with <10^−4^ residual tumor cells showed PFS compared with the rest (not reached (NR) versus 31 months, *P* = 0.002), with similar results observed with MFC. Among complete responders (*n* = 62), PCR discriminated two risk groups with different PFS (49 versus 26 months, *P* = 0.001) and overall survival (NR versus 60 months, *P* = 0.008) [100]. Martinez-Lopez et al. assessed the prognostic value of MRD detection in MM patients using a NGS tool and showed that the applicability of deep sequencing was 91%. Concordance between sequencing and MFC and ASO-PCR was 83% and 85%, respectively. Patients who were MRD– by sequencing had a significantly longer time to tumor progression (TTP) (median 80 versus 31 months; *P* < 0.0001) and overall survival (median not reached versus 81 months; *P* = 0.02), compared with patients who were MRD+ [101]. The conclusion from the above-mentioned studies and many other studies is that MRD assessment in MM using different methods is associated with improvement in PFS and OS which supports the rationale for implementing MRD assessment to redefine and improve current CR criteria in MM [97].

Another way of detecting MRD is by the use of MRI and PET/CT through the detection of possible patchy BM infiltration or extramedullary involvement with an MRD-negative BM [97]. MRI is very sensitive in detecting bone marrow involvement in the spine. PET/CT is able to detect extramedullary disease which has an adverse prognostic impact [102].

A study that compared PET/CT and whole body MRI in transplant-candidate patients showed that, against conventional response criteria, PET/CT had the same sensitivity but higher specificity than whole body MRI [103].

Many studies have shown the value of MRD diagnostics for evaluation of the efficacy of specific treatment stages. Both the Spanish [104] and UK [105] study groups showed the importance of MRD in identifying chemosensitivity before and after ASCT. Failure to eradicate MRD levels before ASCT will show significantly superior PFS if MRD negativity is achieved after ASCT. Another example is a study by Rawstron et al. that showed that patients who achieved MRD negativity with maintenance therapy experienced significantly prolonged PFS [105].

Depth of response was evaluated in different studies. An early study of 126 consecutive patients, of whom 33% achieved CR with SCT, CR did not influence outcome, on either high or low-risk group [99]. Another study at Mayo Clinic showed no significant difference in time to progression (TTP) between the small group of patients who achieved CR before HDT-ASCT (BCR) and those who achieved CR after HDT-ASCT (ACR), at more than 6 years follow-up, and the median OS was not reached at time of analysis [100]. With the advent of novel agents, more CR rates are achieved and its prognostic impact is studied in both relapsed and newly diagnosed MM. Multiple prospective studies of newly diagnosed myeloma patients demonstrated either a longer EFS and/or a better OS in patients who achieved CR or at least VGPR, after a single [101, 102] or tandem ASCT [103–106]. In a large series, including 1000 patients treated with MEL-based tandem high-dose therapy (HDT) trials with autologous hematopoietic stem cell (AHSC) support, superior overall survival was seen in relapsed patients, who achieved a complete remission [107]. However, in a retrospective study which analyzed the outcome of over 500 patients who did not achieve at least a PR after initial induction, there was no difference in median OS between patients who received salvage chemotherapy and those who did not receive any additional therapy to augment response prior to transplant [108]. At this time, we believe that new tools of disease burden are needed to define CR in a more precise way in the era of newer treatments and to study the impact of deeper CR on overall survival.

## 5. Novel Prognostic Markers in MM

Over the past year, there were multiple publications on novel prognostic markers in MM. These include markers in immunophenotyping, genetics, immune signaling, biomarkers, bone marrow environment, and imaging techniques. We selected some of these novel markers.  Table 2 provides summary of some of these promising markers.

## 6. Conclusion and Future Directions

Understanding the prognostic factors in MM is important for optimal care of MM patients.

Our comprehension of the prognostic markers in MM has developed significantly over the last 10 years. Incorporation of different prognostic markers of MM in risk stratification of the disease is evolving with the presence of multiple models in the literature. The ultimate goal of establishing prognostic models in MM is to develop risk-adaptive therapeutic strategies. Our aim of this review is to summarize the current standard prognostic markers used in MM along with novel techniques that are still in development and highlight their implications in current clinical practice. Currently, the most powerful prognostic markers in MM that has clinical implication are genetics abnormalities. The classification of MM into high risk, intermediate risk, and standard risk is based primarily on the impact of genetic aberrations. Selection of therapies nowadays is also directed by this risk stratification. Development of novel prognostic markers is evolving and soon will be part of the standard risk classification in MM and these include GEP and next generation sequencing (NGS).

We believe that refinement of the prognostic models in MM will eventually lead to enhancement of the efficacy of the therapeutic approaches and ultimately will improve the outcome of the disease.

## Figures and Tables

**Table 1:** Various risk stratification models.

Risk stratification model	Prognostic markers	OS	Reference
mSMART	(i) Cytogenetics	(i) Low risk: 10 years	[36]
	(ii) GEP	(ii) Intermediate risk: 4.5 years	
	(iii) PCLI	(iii) High risk: 3 years	

IMWG	(i) ISS (ii) Cytogenetics	(i) Low risk: >10 years	[37]
		(ii) Standard risk: 7 years	
		(iii) High risk: 2 years	

IFM	(i) LDH	Score 0–3. Score 3 had very poor prognosis	[38]
	(ii) ISS		
	(iii) Cytogenetics		

**Table 2:** Summary of some of the novel prognostic markers that were published recently.

Authors	Novel prognostic marker	Conclusion
Li et al. 2015 [39]	The expression patterns of miR-15a/16-1	miR-15a seems to be linked with disease progression and prognosis while miR-16-1 acts as a valuable diagnostic marker
Wang et al. 2015 [40]	Immune checkpoint signaling	The overall response rate to treatment was higher in low sPD-L1 patients than in high sPD-L1 patients
Jung et al. 2016 [41]	Inverse platelet to lymphocyte ratio (iPLR)	Staging by iPLR group had predictive value for PFS and OS
Zhou et al. 2015 [42]	Dysregulated long noncoding RNAs (lncRNAs)	Four lncRNAs were identified to be significantly associated with OS
Lee et al. 2015 [43]	Bone marrow (BM) microvessel density (MVD)	PFS was significantly lower in the high MVD group than in the low MVD group
Ma et al. 2015 [44]	N-Cadherin	OS is worse with high expression of N-Cadherin which may be related to 1q21 amplification.
Lullo et al. 2015 [45]	Th22 cells	Increased frequency of IL-22(+)IL-17(−)IL-13(+) T cells correlates with poor prognosis
Li et al. 2015 [46]	Downregulated miR-33b	miR-33b low expression had significantly shortened PFS and OS
Bolomsky et al. 2015 [47]	Insulin-like growth factor binding protein 7 (IGFBP7) expression	IGFBP7 expression is linked to translocation t(4;14) showing clinical features of adverse prognosis
Jung et al. 2015 [48]	Autophagic markers beclin 1 and LC3	Higher immunoreactivity for autophagic markers in MM is associated with superior patient survival
Trotter et al. 2015 [49]	Myeloma cell-derived Runx2	Runx2 expression is a major regulator of MM progression in bone and myeloma bone disease
